# Head Injury and Associated Sequelae in Individuals Seeking Asylum in the United States: A Retrospective Mixed-Methods Review of Medico-Legal Affidavits

**DOI:** 10.3390/brainsci14060599

**Published:** 2024-06-14

**Authors:** Altaf Saadi, Julia Asfour, Maria Vassimon De Assis, Tessa Wilson, Rohini J. Haar, Michele Heisler

**Affiliations:** 1 Department of Neurology, Massachusetts General Hospital, Boston, MA 02114, USA; 2 Department of Neurology, Harvard Medical School, Boston, MA 02115, USA; 3 School of Arts and Sciences, Tufts University, Boston, MA 02155, USA; jasfour99@gmail.com; 4 School of Public Health, University of California, Berkeley, CA 94704, USA; maria.vassimon@berkeley.edu; 5 Physicians for Human Rights, New York, NY 10018, USA; twilson@phr.org (T.W.); mheisler@med.umich.edu (M.H.); 6 Division of Epidemiology and Biostatistics, School of Public Health, University of California, Berkeley, CA 94704, USA; rohinihaar@berkeley.edu; 7 Department of Internal Medicine, School of Public Health, University of Michigan, Ann Arbor, MI 48109, USA

**Keywords:** refugee health, asylum seekers, migrant health, head injury, head trauma, forensic evaluations

## Abstract

People seeking asylum are susceptible to head injury (HI) due to exposure to various forms of violence including war, torture, or interpersonal violence. Yet, the extents to which clinicians assess HI, and if so, what the associated characteristics are, are not well known. We analyzed 200 U.S.-based medico-legal affidavits using descriptive, multivariate regression, and thematic analysis. Head injury was documented in 38% of affidavits. Those who experienced physical violence were eight times likelier to experience HI than those who did not experience physical violence. Five themes emerged: (1) HI occurred commonly in the context of interpersonal violence (44%), followed by militarized violence (33%); (2) mechanisms of HI included direct blows to the head and asphyxiation, suggesting potential for both traumatic brain injury and brain injury from oxygen deprivation; (3) HI was often recurrent and concurrent with other physical injuries; (4) co-morbid psychiatric and post-concussive symptoms made it challenging to assess neurological and psychiatric etiologies; and (5) overall, there was a paucity of assessments and documentation of HI and sequelae. Among individuals assessed for asylum claims, HI is common, often recurrent, occurring in the context of interpersonal violence, and concurrent with psychological and other physical trauma. Physical violence is an important risk factor for HI, which should be assessed when physical violence is reported.

## 1. Introduction

Some forcibly displaced individuals seek asylum, whereby they can attempt to establish that they have a well-founded fear of persecution if they return to their home countries on the basis of their race, religion, nationality, membership in a particular social group, or political opinion [1]. By nature of their experiences of persecution and forced displacement, people seeking asylum are susceptible to neurological issues like head injury incurred as a result of various forms of violence including war, gang violence, torture, or interpersonal violence [2,3,4]. Exposure to violence broadly, and head injury specifically, can be associated with a range of negative health-related outcomes including mental health diagnoses like depression and anxiety, suicidality, cognitive impairment, and functional impairments such as in employment or social interactions [5,6,7,8].

For forcibly displaced populations like those seeking asylum, head injury can have adverse legal ramifications as well. Head injury leading to memory and other associated cognitive impairments can hinder an individual’s ability to establish credibility as part of their legal case for asylum [9]. Immigration judges and asylum officers rely heavily on an applicant’s testimony—or credibility assessments—to determine if they qualify for asylum status, especially since documentation can often be difficult to provide [10]. During these credibility assessments, immigration judges assess an applicant’s demeanor and responsiveness as well as attempting to identify inconsistencies [11]. Medico-legal examinations and affidavits, based on the guidelines in the Istanbul Protocol—the UN-adopted guidelines for the investigation and documentation of torture and ill-treatment [11]—assess the consistency of physical and/or psychological symptoms and signs with the examined reports of abuse, including an assessment of whether head injury can play a role in an applicant’s testimony or recollection of trauma. These assessments play an important role in helping establish an individual’s claim for asylum. Recent research has found that asylum claims accompanied with a medico-legal affidavit have significantly higher rates of success than those without [12,13].

Globally, traumatic brain injury (TBI) affects over 60 million individuals [14] and is the leading cause of disabilities worldwide in comparison with any other injury [8]. Its high prevalence among forcibly displaced populations is documented alongside the high prevalence of violence suffered and witnessed, but TBI remains under-recognized, partially due to the significant overlap of neurobehavioral post-concussive symptoms and the psychiatric symptoms that are also common in this population [15]. According to one study that included 139 U.S.-based individuals seeking asylum, nearly half self-reported head injury and were more likely to experience headaches and depression than individuals who did not report head injury [16]. A systematic review found a prevalence of TBI of as high as 78%, although prevalence estimates were wide-ranging due to heterogeneity in the studies [15]. Fewer studies in the systematic review explored characteristics of head injury in this population, such as common mechanisms of injury, symptoms, and relationships with psychosocial factors. It is also not known how often head injury is identified and how resulting symptoms and signs are documented in medico-legal affidavits for asylum claims in the United States.

This retrospective study fills this gap by analyzing a national U.S.-based sample of medico-legal affidavits of people seeking asylum obtained from Physicians for Human Rights (PHR) to examine whether clinicians assessed potential head injuries, and if so, how they described head injury characteristics and associated sequelae.

## 2. Materials and Methods

### 2.1. Study Context

Physicians for Human Rights (PHR) is a non-governmental organization based in the United States that documents and advocates against human rights violations by leveraging medical and scientific expertise [17]. PHR’s Asylum Network consists of a network of over 2200 clinicians trained in Istanbul Protocol-based methods who volunteer their time to conduct pro bono forensic medical and psychological evaluations that are used to corroborate the accounts of individuals who are seeking asylum in the United States [18]. The clinicians conduct a formal assessment in which they document past medical and psychiatric history, as well as allegations of incidents of persecution, and conduct a physical and/or psychological examination. Some examinations are conducted by mental health professionals and focus only on psychological symptoms and signs (i.e., forensic psychological evaluations), while some are conducted by physical health specialists such as internists and focus on physical symptoms and signs (i.e., forensic medical evaluations). Many assess both physical and psychological symptoms and signs. Clinicians then compile their findings and describe the consistency of their findings with the alleged abuse in a medico-legal affidavit. These affidavits have been organized into a database by PHR staff over several decades.

### 2.2. Sampling Strategy

Using a random number generator, we selected a sample of 200 of 1346 affidavits collected between 1987 and 2017 in the PHR database. We chose to include affidavits that were (1) physical or psychological evaluations or both, (2) conducted by all types of clinicians (behavioral health, any medical specialty), and (3) for adults and children. If the number generator selected a number that was not affiliated with an affidavit or where the affidavit was missing or excluded, another number was selected. The error in correspondence occurred as not all affidavits within the database included asylum evaluations. All the selected medico-legal affidavits used in this study were de-identified.

### 2.3. Data Extraction

A coding manual was created by the research team to assist with data extraction, informed by the Istanbul Protocol as well as prior PHR coding extraction tools. The coding manual included categories relating to case information, narratives, demographic characteristics, diagnostic data, clinical assessments, and head injury symptom assessments. The coding manual was developed in an iterative fashion, using a small number of affidavits. A pilot test was conducted with 10 affidavits to ensure that the codes were appropriate and that the coded elements of the manual could be populated. With regard to violence, this was categorized as physical violence, rape and sexual assault, witnessed violence, and threatened death or violence, based on an earlier study [2]. Physical violence and rape and sexual assault were directly experienced by the individual. Witnessed violence refers to violence that was experienced by someone else but that the individual observed (i.e., an individual witnessing the killing of a family member). Threatened death or violence refers to the individual being threatened with death or violence (i.e., an intimate partner threatening to kill his wife and their daughter if they run away). An individual could have experienced more than one type of violence. With regard to head injury, the codebook was revised to include head injury both when it was explicitly documented by the evaluator, or if a physical sign on examination suggested there was a head injury (i.e., a scar on the head). We did not apply any formal criteria for TBI; hence, we use “head injury” instead, to distinguish our definition from formal definitions and classifications of TBI. “Head injury” therefore refers to a broad term that describes any trauma to the head, which could include damage to the scalp, skull, brain, and underlying tissue and blood vessels in the head, and may or may not include TBI that results in problems with normal brain function. Head injury “sequelae” refers to post-concussive symptoms, which can include somatic symptoms (e.g., nausea, dizziness, headache, fatigue), cognitive complaints (e.g., memory and executive function), and/or emotional problems (e.g., disinhibition, emotional lability, irritability). Using the coding manual, two independent research assistants read through every affidavit to identify and extract relevant information. Interrater reliability was measured using Cohen’s Kappa, which was high (k > 0.85).

### 2.4. Statistical Analysis

We performed a descriptive analysis to characterize the descriptive data and describe the relationship among demographic variables, case-specific variables, and the head injuries documented by the clinicians. Group comparisons were conducted using a *t*-test for continuous variables and Pearson’s χ^2^ for categorical variables in univariate analyses. Multivariable logistic regression was performed to examine the association between head injury and physical violence, and 95% confidence intervals (CIs) for crude and adjusted odds ratios (ORs) were calculated. Odds ratios were adjusted for clients’ age and sex. A *p*-value of <0.05 was considered statistically significant. We used Stata/SE15.1 to analyze our data.

### 2.5. Qualitative Analysis

We used the framework method [19], which involved a hybrid inductive–deductive approach to interpreting our data, to determine the most salient themes among the 75 affidavits that involved head injury, to understand the nuances and complexities of head injury-related symptoms and signs in this population beyond patterns noted in the quantitative analysis. This was an iterative process, involving code merging and reconciliation at regular team meetings. A coding scheme across five themes emerged through consensus.

### 2.6. Ethics

This study was approved and deemed exempt by the Institutional Review Board (a blinded for peer review) and by the Ethics Review Board (a blinded for peer review).

## 3. Results

In this sample (*N* = 200), the average age of evaluated individuals was 32 ± 13 SD (range 4–75). In total, 38% (*n* = 75) had documented head injury. There were 16 individuals who were < 18 years old and only four who had documented head injury. Although most of the individuals in the entire sample identified as female (53%, *n* = 105), the majority of those whose medico-legal affidavit had an identified head injury were male (57%, *n* = 42). Table 1 describes the sociodemographic and clinical characteristics of the sample, including differences between those with and without head injury. Those with head injury had more frequently suffered physical violence than not and had a more documentation of loss of consciousness. In this sample, there were more psychological forensic evaluations (79%, *n* = 157) than physical evaluations (52%, *n* = 104), but head injury was more frequently documented in a physical evaluation than in a psychological evaluation (*p* = 0.003).

Table 2 describes the most common characteristics of head injury documented in the medico-legal affidavits, which we integrate into a presentation of qualitative themes below. In assessing the association between head injury and physical violence experienced in the sample, we found that those who reported experiencing physical violence had nearly eight-fold higher odds of reporting head injury (AOR = 7.7, 95% CI = 3.5, 16.9, *p* ≤ 0.0001) than those who did not report experiencing physical violence. Adjusted odds ratios were controlled for age and gender. This association was not seen among those who witnessed violence or threatened death/violence.

We identified five major themes relating to head injury in this sample of individuals seeking asylum in the U.S.: (1) head injury occurred most commonly in the context of interpersonal violence, followed by militarized violence, which we use to refer to both state-sponsored violence and violence perpetrated by non-state armed groups or local gangs; (2) mechanisms of head injury included both direct blows to the head and asphyxiation, suggesting potential for both TBI and brain injury from oxygen deprivation; (3) head injury was recurrent and concurrent with other physical injuries; (4) co-morbid psychiatric and post-concussive symptoms made it difficult to disentangle neurological and psychiatric etiologies; and (5) overall, there was a paucity of assessments and documentation of head injury incidence and sequelae. These themes, describing the experiences of individuals seeking asylum, are elaborated below via descriptive statistics (Table 1 and Table 2) and illustrative quotes.

### 3.1. Head Injury Occurred Most in the Context of Interpersonal Violence, Followed by Militarized Violence

The reviewed affidavits described a range of contexts where head trauma, among other violent acts, was perpetrated. Twenty percent (*n* = 15) of the affidavits were of individuals who reported intimate partners as perpetrators of head injury. While speaking to a 51-year-old woman from Honduras who was a survivor of domestic abuse, a clinician reported a “20-year history of abuse” that involved beating “*her with his fists but also whatever blunt object happened to be handy*” resulting in cognitive deficits that were highlighted by a Mini Mental State Exam, where “*she scored a 20 out of a maximum of 30*” and “*raised the clinician’s suspicion for TBI*”. This woman was also raped, which was a common co-occurring reality for female survivors of intimate partner violence and HI.

Interpersonal violence was not limited to intimate partner violence, however. Twenty one percent (*n* = 16) of the affidavits reported family members, ranging from immediate to extended family, as perpetrators of head injury. One woman from China was documented to experience the following: “*her father pushed her against a wall and causing blunt trauma to the right side of her forehead*”. Another man, a 19-year-old from Honduras, was documented to experience head injury because of violence from an aunt: “*At least once daily she [his aunt] hit him with her hand or pulled on his ears… His aunt often hit him with sticks and larger pieces of wood, threw rocks at him, and whipped him on his back with ropes or electrical or metal wires. He recalls losing consciousness 2 or 3 times … Once he ran away from his aunt when she was throwing rocks at him, but he fell and lacerated his forehead on a pipe that he was sticking out of the ground*”.

Violence from family members or even community members resulting in head injury was particularly prevalent among many LGBTQ+ individuals. According to medico-legal affidavits, a 26-year-old man from Honduras reported being “*periodically threatened and assaulted because other boys thought he was gay*”. According to the affidavit, “*His father tried to restrict his movements by locking him into his room with a chamber pot while he was at work; and he even took him to visit prostitutes to cement a mistaken heterosexuality. His father hit him over the head with a pipe wrench on another occasion*”.

Violence by state actors, non-state armed groups, or local gangs was a frequent occurrence, with head injury by these perpetrators documented (we defined these cases collectively as “militarized violence” for simplicity). According to one medico-legal affidavit involving a 45-year-old man from Mauritania, “*During his detainment by the police…he was stripped naked, hung upside down and the soles of his feet were repeatedly beaten with a wooden stick... he was buried up to his neck in sand, threatened with death and kicked in the head by his captors when he eventually lost consciousness*”.

### 3.2. Causes of Head Injury Included Both Direct Blows to the Head and Asphyxiation, Suggesting Potential for Both TBI and Brain Injury from Oxygen Deprivation

The principal mechanisms of head injury were direct head strike caused by an external force, as well as asphyxiation or strangulation, which can cause brain injury via oxygen deprivation or alterations in consciousness (AICs). According to the medico-legal affidavit of a 47-year-old woman from Nigeria, for example, “*During her third pregnancy, her husband came home late one night and had an argument with her and held her down and choked her almost to the point of losing consciousness*”. She reported head injury from both mechanisms of injury: “*[She] report[ed] many instances of physical abuse inflicted upon her by her husband… he would kick her, choke her, and use his hands and fists to strike her in the head. Sometimes he would also use his belt to hit her*”.

Another 51-year-old woman from Honduras reported that she “*had received head beatings [from her partner] to the point of unconsciousness. She had also been suffocated and starved to the point of near death*”.

Many victims reported direct head strikes with blunt objects. A 30-year-old man from Somalia was detained by Ethiopian soldiers and reported that they “*hit him on his head with [a] wood handle*”. Additionally, he was “*hit with the butt of a gun on his head, shoulder, and back of his neck*”. A 26-year-old man from Honduras reported that his father “*threw a lit gas lamp at him once and hit him over the head with a pipe wrench on another occasion*”.

### 3.3. Head Injury Was Recurrent and Concurrent with Other Physical Injuries

Clinicians identified that individuals sustained multiple injuries across differing contexts. For some, these injuries resulted in a loss of consciousness and memory loss or even extra-cranial injuries. Among those who sustained head injury, 41% (*n* = 31) reported multiple head injuries. One woman, a 30-year-old from Djibouti, “*recalls being struck multiple times in the head [by her husband]. She was mostly struck in the temple and ear regions*”. She was also noted to have “*chronic headaches due to repeated blows to the head*” that additionally resulted in ruptured ear drums. Other physical symptoms or injuries included ringing in the ears or hearing impairment, blurry vision or other visual symptoms from orbital injury, spinal injury, peripheral nerve damage, and musculoskeletal injuries.

### 3.4. Co-Morbid Psychiatric and Post-Concussive Symptoms Made It Difficult to Disentangle Neurological and Psychiatric Etiologies

Individuals with head injury reported sleep difficulties (64%, *n* = 48) and headaches (44%, *n* = 33) as the most common symptoms that are well documented post-concussive sequelae. For example, one 45-year-old man from Mauritania who had suffered head injury from being kicked in the head reported “*trouble falling asleep in the evenings*”. Another man, a 29-year-old from Rwanda, had suffered head injury from a bayonet hit against his head by the Rwandese military was documented to experience “*great difficulty falling asleep. He describes that he sleeps four hours at most and sometimes none at all*”. With regard to headaches, a 40-year-old man from the Democratic Republic of the Congo reported “*headaches following the trauma to his head*” that involved being struck on the head with the butt of a rifle and losing consciousness.

However, distinguishing between whether psychiatric symptoms were a consequence of, or comorbid with, head injury was not possible in many cases given the co-occurrence of traumatic experiences. This was particularly true for cognitive complaints. For example, one 42-year-old woman from Guatemala had her head slammed into the floor and kicked repeatedly by her husband reported “*difficulty with focus*” but was also documented to have “*psychiatric symptoms (suicidal ideation, anorexia, psychosomatic symptoms) consistent with a more severe level of trauma, depression, and anxiety*” that made it difficult to distinguish the underlying mechanism. While speaking to a 65-year-old woman from the Democratic Republic of the Congo who had suffered head injury from being kicked and hit on the head with a rifle butt, a clinician reported that she had post-traumatic stress and depressive symptoms that overlapped with post-concussive symptoms including memory problems and depressive mood.

### 3.5. Paucity of Assessments and Documentation of Head Injury and Traumatic Brain Injury Incidence and Sequelae

Thirty percent (*n* = 58) of affidavits documented head injury as disclosed by the applicant, and an additional 8.5% (*n* = 17) cases documented scars involving the head, suggesting head injury, in which the clinician did not then seek to determine from the individual being examined whether or not the scar resulted from a head injury during the alleged abuses. None of the affidavits in this sample utilized a standardized TBI assessment tool. Moreover, no full neurological exam was completed or documented in any affidavit, even in those in which the individual reported head trauma. Just one affidavit documented a partial neurological exam including cranial nerve testing and finger-to-nose testing to assess for cranial nerve injury and cerebellar function, respectively. Even when physical sequelae such as headache or cognitive impairment that could be due to head injury were documented, they were not explored in depth. This was even the case among clients who self-disclosed head injury that involved loss of consciousness.

Forty one percent (*n* = 31) of people with head injury reported loss of consciousness, though this detail was not consistently reported among those with head injury. Further, post-traumatic amnesia, which can be used to determine TBI severity alongside the duration of loss of consciousness, was not elicited by any of the clinicians in the medico-legal affidavits. Consequently, not only were no standardized TBI assessment tools used, but clinicians also did not gather pertinent details to diagnose TBI or its severity. None of the medico-legal affidavits mentioned use of brain imaging.

## 4. Discussion

Our study characterizes the documentation of the occurrence, characteristics, and assessed sequelae of head injury in a national sample of medico-legal affidavits used for asylum claims of 200 individuals seeking asylum in the United States. Our findings highlight the high prevalence of head injury when an individual has endured any physical violence and the recurrent nature of head injury in this population. The major mechanisms of injury were direct strikes, but also included strangulation-related head injury. Head injuries occurred both in cases of intimate partner violence and violence from other family members. Yet, as both physical and psychological trauma manifest with a significant overlap between symptoms and signs, it was often not possible to disentangle etiologies. Overall, we found significant room for improvement in the assessment of head injury and its sequelae in this population and in medico-legal affidavits—including the need for the use of standardized screening tools and for training in identifying traumatic brain injury (TBI).

Although nearly 40% of the sample had documented head injury, this likely represents an underestimate due to the lack of standardized screening. Another study of individuals seeking asylum in Miami similarly reported that 43% in their sample explicitly self-reported history of injury [16], but a systematic review found a wide range across studies, as high as 78%, depending on how head injury history was elicited [15]. Some of the reasons for not proactively disclosing head injury include shame, stigma, and the lack of awareness of available resources or lack of awareness that head injury might be relevant to the asylum claim [15,20]. It is further suggestive of under-reporting that nearly 10% of head injury cases were not explicitly described by individuals in the trauma narrative but rather identified upon physical examination via the documentation of scars involving the head. A more systematic and specialized approach in forensic medical and psychological evaluations for asylum seekers could overcome barriers to voluntary self-disclosure. In the context of intimate partner violence (IPV), for example, violence may become so normalized that the victim stops registering certain violent incidents as significant or worthy of disclosure. Others, including children, may have blocked traumatic episodes from memory and need sufficient time to uncover trauma with a mental health professional before being able to disclose it. Some are also conditioned not to disclose IPV or family violence for fear of retaliation by the perpetrator. Since head injury was eight times more likely to have occurred than to have not occurred in cases of reported physical violence, it is essential for clinicians conducting forensic medical evaluations to universally assess for head injury in these cases. In addition, lawyers also need to ask their clients about head injuries when eliciting the forms of harm suffered, to alert clinicians about the possibility or presence of head injury in medical–legal partnerships involving forensic medical evaluations. On the part of asylum adjudicators, they can reasonably assume that a significant portion of individuals seeking asylum have sustained a head injury, given the pervasiveness of physical violence in this population and our study findings, even if someone has not received a formal medical evaluation. This is especially important as head injuries can lead to cognitive impairment that can affect individuals’ apparent credibility in asylum adjudications if not considered or accounted for, as well as negatively impacting social relationships and opportunities for employment [21]. Recurrent head injuries, which we also found in our sample, can further increase the risk of these health and social harms.

We further found that head injury includes multiple mechanisms of injury—in our sample universally involving direct head strike, but also brain injury from strangulation or asphyxiation. Strangulation has been linked to impairments in cognitive and psychological function [22]. In one study involving women survivors of IPV, those who had experienced strangulation-related alterations in consciousness performed worse on a test of long-term memory than women who had not experienced strangulation-related alterations in consciousness, even after accounting for IPV-related traumatic brain injuries [22]. They also had higher levels of depression and post-traumatic stress symptomatology. This reinforces the importance of adapting existing screening measures to include questions about strangulation or choking.

Our finding that head injury in this population was reported in cases of interpersonal violence more broadly, rather than IPV alone, is important as it encourages a more expansive view of who may be impacted by head injury. This is particularly applicable for LGBTQ+ individuals who have a higher reported incidence of sexual violence [23]. The results of one New York-based study of individuals seeking asylum showed that approximately 70% of the LGBTQ+ participants had experienced persecution in the form of sexual violence [23]. Among this population, mental health illnesses, PTSD, and suicidal ideation were also highly prevalent. Of the study participants, 46% of the LGBTQ+ participants had been persecuted by family members. This highlights the risk individuals may face within familial structures.

We found that when head injury was documented, it was more likely to be reported in forensic medical evaluations conducted by clinicians from physical medical specialties (e.g., internal medicine, neurology, family medicine) than psychological evaluations conducted by psychiatrists, psychologists, or licensed clinical social workers. This reinforces the importance of targeted educational interventions that discuss the overlap between post-concussive and psychological symptoms and that specifically include clinicians conducting psychological evaluations. Psychological symptoms or diagnoses can independently contribute to memory challenges [2] or complicate recovery following TBI, such as by contributing to persistent post-concussive symptoms, and both would be important in the forensic immigration context where such symptoms can hinder people’s ability to establish credibility. In particular, just as clinicians often routinely use validated instruments to screen for psychological symptoms such as the PTSD Checklist for DSM-5 (PCL-5) for PTSD, or the Patient Health Questionnaire (PHQ-9) for depression and General Anxiety Disorder-7 (GAD-7) for anxiety, clinicians should always ask specifically about head injuries, especially when physical violence is reported. They should also use validated instruments to screen for TBI and other sequalae of head injuries when screening for head injury is positive, including but not limited to the Ohio State University TBI Identification Method or the Brain Injury Screening Index [24,25]. Further, educational interventions must discuss when additional testing such as brain imaging or targeted neurological or neuropsychological assessments would be appropriate, if a clinician does not feel comfortable with their own assessment. It is heartening that the Asylum Medicine Training Initiative, founded in 2021 to train healthcare professionals to meet the need for forensic medical evaluations of people seeking asylum in the US, includes a module on TBI [26]. Future attention to neuropsychiatric issues in this population should also integrate the high prevalence of headaches and sleep disturbances we identified in our study, which could represent post-concussive symptoms or be comorbid with a co-occurring psychological illness like PTSD and depression, which require independent attention and treatment [27,28].

We recognize that not all immigrants are able to access forensic medical evaluations and acknowledge concerns raised by legal practitioners about overly stringent evidentiary standards that require immigrants to gain access to health professionals to obtain a positive immigration outcome [29]. As such, a better understanding among health care providers of how head injuries can impact a victim’s ability to recount trauma must be coupled with more effective and comprehensive education of immigration adjudicators. Given the high prevalence of physical violence in this population and the eight-fold increase in head injury among those who have suffered physical violence, our findings suggest that adjudicators should consider the possibility of head injury and TBI when making credibility decisions, even if forensic medical evaluations are not present.

## 5. Limitations

This study has several limitations. First, our analysis was of individuals seeking asylum who were represented by lawyers who requested medico-legal assessments and thus does not represent those who were not represented by lawyers and unable to receive an assessment. The pre-screening by legal teams who determined that a medico-legal affidavit would be helpful might mean this sample had a higher chance of having experienced physical and/or psychological trauma than other individuals seeking asylum, including head injury. While this may mean that the study may have overestimated the prevalence of head injury, other studies have suggested that its prevalence is underestimated and underdiagnosed due to various factors like a lack of standardized and universal screening, and stigma and shame [15, 20]. This underscores the need for future studies that assess TBI in a systematic manner in this population, using validated tools. Second, our sample was drawn from affidavits written between 1987 and 2017, and thus the included affidavits may not be typical of affidavits written before or after this period. Third, there may be unique considerations in pediatric populations that require further focused study. Despite the inability to generalize the findings from this sample, our findings from the examined affidavits provide important insights into key challenges in screening for, considering, and documenting head injuries and their sequelae in a highly trauma-exposed population.

## 6. Conclusions

In summary, while our review found high rates of head injury, it highlighted the need for improved screening and assessment, as well as the more effective and comprehensive education of clinicians caring for this population and conducting forensic medical and psychological evaluations, as well as if immigration adjudicators. Future studies would benefit from prospective data collection and from standardized screening for head injury and TBI using validated tools.

## Figures and Tables

**Table 1:** Sociodemographic characteristics of the total sample.

	Total Sample (*N* = 200)	No Head Injury (*n* = 125)	Head Injury (*n* = 75)	*p*-Value
**Age** (mean ± SD)/193 **	32.4 (12.7)	31.8 (12.5)	33.5 (12.9)	0.36
**Gender** (*N*, %)				0.031 *
Male	95 (48%)	52 (42%)	43 (57%)	
Female	105 (53%)	73 (58%)	32 (43%)	
**Type of Evaluation** (*N*, %)				
Psychological	157 (79%)	103 (82%)	54 (72%)	0.126
Physical	104 (52%)	55 (44%)	49 (65%)	0.003 *
**Type of Violence** (*N*, %)				
Physical Violence	126 (63%)	61 (49%)	65 (87%)	<0.001 *
Rape and Sexual Assault	71 (36%)	45 (36%)	26 (35%)	0.85
Witnessed Violence	76 (38%)	51 (41%)	25 (33%)	0.29
Threats of Death or Violence	136 (68%)	84 (67%)	52 (69%)	0.754
**Perpetrator of Violence** (*N*, %)				
Interpersonal Relationship	89 (45%)	55 (44%)	34 (38%)	0.85
Friend or Community members	27 (14%)	17 (14%)	10 (13%)	0.96
Family	44 (22%)	28 (22%)	16 (21%)	0.86
Intimate Partner	40 (20%)	25 (20%)	15 (20%)	1.0
Police, Military, Prison Guards	63 (32%)	38 (30%)	25 (33%)	0.67
Gang members	39 (20%)	25 (20%)	14 (19%)	0.82
**Neurological Characteristics** (*N*, %)				
Loss of Consciousness	44 (22%)	13 (10%)	31 (41%)	<0.001 *
**Psychological Illness** (*N*, %)				
Depression	113 (56%)	79 (63%)	34 (45%)	0.014 *
Anxiety	35 (18%)	19 (15%)	16 (21%)	0.27
PTSD	138 (69%)	91 (66%)	47 (34%)	0.13

* *p* < 0.05 (χ^2^ for proportions; two-sided *t*-tests for averages). ** The denominator varies from the total sample due to missing data.

**Table 2:** Common characteristics of head injury in subsample (*n* = 75).

	(*N*, %)
**Loss of Consciousness**	31 (41%)
**Multiple Head Injuries**	27 (36%)
**Mechanism of Injury**	
Blunt Trauma	75 (100%)
Asphyxiation (strangulation, drowning)	3 (4%)
**Signs and Symptoms**	
Sleep Dysfunction	46 (61%)
Headache	31 (41%)
Anger/Irritability	14 (19%)
Cognitive Challenges	21 (28%)
Vision changes	9 (12%)
Hearing Changes	13 (17%)
Physical Scar in Head Region	30 (40%)

## Data Availability

Data may be requested by reaching out to Physicians for Human Rights’ Tessa Wilson at twilson@phr.org. Due to the lack of consent and the sensitivity of the data, which include data on minors, it would not be ethical, or legal, for PHR to widely release these client data. PHR’s Ethics Review Board is able to field requests for the use of these data on an individual basis.
